# Impact of Modality and Intensity of Early Exercise Training on Ventricular Remodeling after Myocardial Infarction

**DOI:** 10.1155/2020/5041791

**Published:** 2020-07-20

**Authors:** Diego Fernando Batista, Bertha Furlan Polegato, Renata Candido da Silva, Renan Turini Claro, Paula Shmidt Azevedo, Ana Angélica Fernandes, Katashi Okoshi, Sergio Alberto Rupp de Paiva, Marcos Ferreira Minicucci, Leonardo Antônio Mamede Zornorff

**Affiliations:** ^1^Internal Medicine Department, Botucatu Medical School, São Paulo State University (UNESP), Botucatu, Brazil; ^2^Chemistry and Biochemistry Department, Institute of Biosciences of Botucatu, São Paulo State University (UNESP), Botucatu, Brazil

## Abstract

The objective of this study was to analyze the impact of different modalities and intensities of exercise training on cardiac remodeling started early after experimental myocardial infarction (MI). Male Wistar rats, weighing 200–250 g, were subjected to experimental MI. After 5 days, the animals were allocated into three experimental groups and observed for three months: S (sedentary control animals), C (animals subjected to continuous low-intensity training), and HIT (animals subjected to high-intensity interval training). Low-intensity exercise training was performed at a treadmill speed corresponding to 40% VO_2_ max, which was kept unchanged throughout the entire session (i.e., continuous low-intensity training). High-intensity interval training was performed in such a way that rats run during 3 min at 60% VO_2_ max, followed by 4-minute intervals at 85% VO_2_ max (i.e., high-intensity interval training). After the follow-up period, we studied hypertrophy and ventricular geometry, functional alterations *in vivo* and *in vitro*, oxidative stress, apoptosis, and cardiac energetic metabolism. Our data showed that both high-intensity interval and continuous low-intensity modalities improved cardiac energetic metabolism variables in comparison with sedentary infarcted animals. In addition, high-intensity interval training decreased cardiac oxidative stress, associated with improved diastolic function. On the other hand, the continuous low-intensity group showed impairment of cardiac function. Therefore, altogether, our data suggest that high-intensity interval training could be the best modality for early physical exercise after MI and should be better studied in this clinical scenario.

## 1. Introduction

After myocardial infarction (MI), ventricular remodeling is associated with poor outcomes, mainly due to increased risk for ventricular dysfunction and cardiovascular death. Therefore, strategies to attenuate this process are fundamental tools in the management of patients with coronary occlusion, including reperfusion therapy, beta-blockers, and inhibitors of the renin-angiotensin system [1–4].

Exercise training started late after experimental MI is accepted as a strategy to attenuate the remodeling process [5–9]. However, the best time to start a training program after a coronary occlusion remains to be elucidated. Indeed, during the healing phase, a number of factors may stimulate the progressive dilation of the heart by increasing ventricular wall stress. Importantly, it is postulated that early exercise might act as a parietal stressor, increasing the left ventricular dimensions [1]. In fact, in a previous study, our group showed that delayed exercise might be better than early exercise following coronary occlusion [10].

We must consider, however, that the remodeling process starts at very early stages after coronary occlusion. Thus, strategies initiated early could have greater repercussions in mitigating ventricular remodeling after MI. In addition, in recent years, new modalities of physical exercise have been introduced in different models with promising results, including interval training. Importantly, it remains unknown if modality and intensity of early exercise training, after MI, could also influence the remodeling process. Therefore, the objective of this study was to analyze the impact of different modalities and intensities of exercise training on cardiac remodeling started early after experimental MI.

## 2. Material and Methods

All experiments and procedures were performed in concordance with the National Institute of Health's Guide for the Care and Use of Laboratory Animals and were approved by the Animal Ethics Committee of our institution.

### 2.1. Experimental Groups

**Figure 1:**
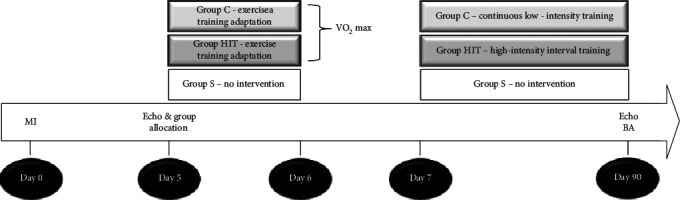
Experimental timeline. MI: myocardial infarction; echo: echocardiogram; VO_2_ max: maximum oxygen uptake; BA: biochemical analysis.

The experimental timeline is shown in Figure 1. Male Wistar rats, weighing 200-250 g, were subjected to experimental MI, according to the method described previously [11, 12]. We selected only the animals with an infarct size greater than 35% as assessed by histologic analysis because we considered that animals with small infarct size do not undergo cardiac remodeling [13].

After 5 days of performing the surgical procedure of infarction induction, an initial echocardiographic study was performed to evaluate systolic and diastolic areas, fraction area changes, and infarct size, to guarantee homogeneity between the groups (data not shown).

After the initial echocardiographic study, the animals were allocated into three experimental groups and observed for three months: S (sedentary control animals), C (animals subjected to continuous low-intensity training), and HIT (animals subjected to high-intensity interval training).

### 2.2. Exercise Training Protocols

Exercise training protocols started 5 days after surgical procedures. The speed started at 6 m/min and was increased by 3 m/min every 3 min until the rats could not run. The same protocol was also used to measure the maximum oxygen uptake (VO_2_ max). The animals were placed inside a metabolic chamber (airflow inside the chamber (3.500 mL/min)). Low-intensity exercise training was performed at a treadmill speed corresponding to 40% VO_2_ max, which was kept unchanged throughout the entire session (i.e., continuous low-intensity training). High-intensity interval training was performed in such a way that rats run during 3 min at 60% VO_2_ max, followed by 4-minute intervals at 85% VO_2_ max (i.e., high-intensity interval training), which was repeated seven times, so each HIT session lasted for 49 min. Similar to continuous low-intensity training, this protocol was kept unchanged throughout the entire session. Continuous low-intensity and HIT protocols were of matched volume, meaning that total running distances in each session of either continuous low-intensity training or HIT were identical [14]. The protocols were performed at 15° inclination, 5 days per week, over 3 months.

### 2.3. Echocardiographic Analysis

At the end of the three-month follow-up, echocardiography was performed. The cardiac structures were measured according to previous methods in an infarcted rat model [15, 16]. The systolic (SA) and diastolic areas (DA) were measured in two dimensions through planimetry. The left ventricle (LV) function was assessed by calculating the fractional area change (FAC = (DA − SA)/DA × 100), the posterior wall shortening velocity (PWSV), the Tei index, the *E* wave deceleration time (EDT), the isovolumetric relaxation time normalized to the heart rate (IRT/RR^0.5^), and the *S*′ wave and *A*′ wave, assessed by tissular echocardiogram.

### 2.4. In Vitro Left Ventricular Function Analysis

The procedures and measurements for assessing left ventricular function were performed following a previously described method [17]. The LV systolic function was assessed by the maximum systolic pressure (PS) and the maximum rate of ventricular pressure rise (+dP/dt). The LV diastolic function was assessed by the decreased maximum rate of ventricular pressure rise (-dP/dt).

### 2.5. Cardiac Energy Metabolism and Oxidative Stress

By spectrophotometry, LV samples of approximately 200 mg were homogenized in sodium phosphate buffer (0.1 M, pH 7.0) and centrifuged at 10,000 rpm for 15 minutes at 4°C. The supernatant was used to determine the concentrations of protein, lipid hydroperoxide, and antioxidant enzymes and energy metabolism. The activities of the enzymatic complexes of the mitochondrial respiratory chain were determined after resuspension of the pellet in phosphate buffer containing 0.1 M sodium, 250 mM sucrose, and 2 mM ethylenediaminetetraacetic acid (EDTA) and centrifugation (10,000 rpm, 5 minutes, 4°C) according to an adapted technique [18].

The activities of lactate dehydrogenase (LDH), citrate synthase, *β*-hydroxyacyl-CoA dehydrogenase (*β*-OH-acyl-CoADH), phosphofructokinase (PFK), pyruvate dehydrogenase complex (PI-DH), NADH dehydrogenase (complex I), succinate dehydrogenase (complex II), and ATP synthase were measured by the method described previously [18].

Oxidative stress was assessed by determining the concentration of lipid hydroperoxide (LH). Glutathione peroxidase (GSH-Px) and catalase (CAT) activities were assessed as previously specified [19].

### 2.6. Statistical Analysis

The data were expressed as mean ± standard deviation (for normal distribution) or median with the 25th and 75th percentiles (for nonnormal distribution). Continuous variables were tested for normality; continuous variables with normal distribution were compared by ANOVA, completed by the Holm-Sidak test, while nonnormal continuous variables were compared by Kruskal-Wallis test, completed by the Dunn test. Data analysis was performed with SigmaStat for Windows v2.03 (SPSS, Inc., Chicago, IL, USA). The significance level was 5%.

## 3. Results

We subjected 140 animals to coronary occlusion. After 5 days, 50 rats died. In addition, 20 animals were discarded for having an infarct size of <35%. Therefore, our groups were composed of the following animals: S = 25, C = 25, and HIT = 22.

There were no differences in body weight between the groups at the beginning of the experiment (S = 232 ± 28 g, C = 226 ± 18 g, and HIT = 229 ± 15 g; *P* > 0.05) or after 3 months (S = 504 ± 39 g, C = 483 ± 54 g, and HIT = 459 ± 11 g; *P* > 0.05).

There was no difference in infarct size between the groups (S = 47 ± 3.6%, C = 46 ± 4.2%, and HIT = 48 ± 2.5%; *P* > 0.05). Likewise, in the period of 3 months after the infarction, there was no difference in the mortality between the groups (S = 10, C = 7, and HIT = 7; *P* > 0.05).

The results of the echocardiographic study are shown in Table 1. The C group presented higher values of EDT than the HIT group and higher values of the Tei index than the S group. In addition, the C group presented higher values of IRT/RR^0.5^ in comparison to groups HIT and S. The animals of the HIT group presented higher values of *A*′ than the other groups. There was no difference in the other variables between the groups. Likewise, in the study of the isolated heart, we did not find differences among the groups (Table 2).

In relation to the results of oxidative stress, the animals of the HIT group presented higher values of the antioxidant enzymes catalase and glutathione peroxidase. As a consequence, intense interval exercise showed a decrease in oxidative stress, assessed by LH concentrations. Importantly, this phenomenon did not occur with the group of animals subjected to continuous exercise (Table 3).

Considering the data on energy metabolism, both trained groups presented a decrease in lactate dehydrogenase activity and increased ATP synthase and mitochondrial complex I activity. On the other hand, only the HIT group showed increased activity of *β*-hydroxyacyl coenzyme-A dehydrogenase, citrate synthase, and pyruvate dehydrogenase (Table 4).

## 4. Discussion

The objective of this study was to analyze the impact of different modalities and intensities of exercise training on cardiac remodeling started early after experimental MI. Our data showed that both high-intensity interval and continuous low-intensity modalities improved cardiac energetic metabolism variables in comparison with sedentary infarcted animals. In addition, high-intensity interval training decreased cardiac oxidative stress, associated with improved diastolic function. On the other hand, the continuous low-intensity group showed impairment of cardiac function.

As discussed earlier, the beneficial effects of late physical training after MI are unquestionable [5–9]. On the other hand, when physical exercise is started early after coronary occlusion, the results in the remodeling process are not uniform. In rats with large infarctions of the anterior wall, physical exercise started less than one week after coronary occlusion resulted in an increase in infarct expansion with dilation of the left ventricular cavity. The authors hypothesized that early physical exercise could increase parietal stress and stimulate the remodeling process [20–23]. In contrast, in mice and rats, nonintense physical exercise started one week after infarction of different sizes attenuated the remodeling process or had no morphological repercussion [24–27]. Therefore, we can conclude that the effects of physical exercise after infarction depend on different variables, including infarct size, and the exercise protocol used. Importantly, the role of modality and intensity of early exercise training in ventricular remodeling after MI remains to be elucidated.

Our study showed that high-intensity interval training improved diastolic function. On the other hand, the continuous low-intensity group showed impairment of cardiac function, assessed by the Tei index and IRT/RR^0.5^ ratio. Importantly, early exercise after MI did not improve systolic function. We have to consider that the beneficial effects of exercise initiated late after the experimental infarction are consensual, including improved systolic function. Therefore, our data reinforce the concept that, at least so far, late physical exercise protocols after infarction could be better than early physical exercise, regardless of the modality or intensity.

Reactive oxygen species and oxidative stress play a critical role in cardiac remodeling following several cardiac injuries [28, 29]. To protect against oxidative stress, a well-organized system of antioxidants works in a coordinated manner. In our study, both modalities increase antioxidant enzymes, but only the high-intensity interval training protocol decreased oxidative stress, assessed by LH. Therefore, considering the redox system, we can conclude that HIT was superior to continuous low-intensity training.

Another potential mechanism involved in the action of exercise training on cardiac remodeling is related to cardiac energy metabolism [30]. The remodeled heart exhibits several changes in energy metabolism, including a fuel preference shift, decreased fuel amount, mitochondrial abnormalities, and impaired transport of energy from mitochondria to the site of utilization. Data on energy metabolism suggest that, despite some specific differences, in general, high-intensity interval training increased the energy substrate and mitochondrial phosphorylation activity, more evident than continuous low-intensity training.

Finally, our data showed that the evident benefits in oxidative stress and cardiac energy metabolism in the high-intensity interval training group did not result in improved systolic function, which is a critical variable in the assessment of cardiac remodeling. However, we must understand that the temporal evolution of the remodeling process could explain these apparently discrepant results. In fact, the first event in this process is a stimulus to the heart, including hemodynamic overload, loss of myocytes, cardiac inflammation, genetic changes, and toxic injuries. As a consequence, fetal genes are reexpressed. This phenomenon, in turn, results in deleterious cardiac responses in different pathways, including inflammatory systems, cell death, collagen accumulation, energy metabolism deficit, and oxidative stress. Finally, as a result of these events, the remodeling process is associated with changes in cardiac morphological and functional variables [1–4]. Therefore, with a longer follow-up time, we believe that the beneficial biochemical changes induced by high-intensity interval training in this study would result in cardiac systolic functional improvement.

In conclusion, both high-intensity interval and continuous low-intensity modalities improved cardiac energetic metabolism variables. In addition, the high-intensity interval modality decreased cardiac oxidative stress, associated with improved diastolic function. On the other hand, a continuous low-intensity training group showed impairment of cardiac function. Therefore, altogether, our data suggest that high-intensity interval training could be the best modality for early physical exercise after MI and should be better studied in this clinical scenario.

## Figures and Tables

**Table 1:** Echocardiographic data.

Variables	S (*n* = 15)	C (*n* = 16)	HHIT (*n* = 15)
LVDD (mm)	10.4 ± 0.9	10.6 ± 1.1	10.3 ± 1.2
LVSD (mm)	7.85 ± 1.0	8.21 ± 1.4	8.53 ± 0.2
EF (%)	0.57 ± 0.1	0.53 ± 0.1	0.50 ± 0.1
FAC (%)	36.5 ± 11.1	30.7 ± 13.4	33.9 ± 14.5
Tei index	0.56 (0.47-0.65)	0.69 (0.59-0.84)^∗^	0.60 (0.55-0.66)
LVWT (mm/s)	29.7 ± 5.7	19.3.±6.7	27.9 ± 7.4
EDT	43.3 ± 7.6	47.4 ± 8.7	37.3 ± 10.8^#^
IRT/RR^0.5^	57.3 ± 8.8^#^	66.2 ± 9.1	57.2 ± 14.3^#^
*S*′	3.10 ± 0.48	2.79 ± 0.35	3.09 ± 0.56
*A*′	3.29 ± 1.19	3.46 ± 0.86	4.69 ± 1.15^∗^^#^

S: sedentary control animals; C: continuous moderate intensity training; HIT: high-intensity interval training; LVDD: left ventricular diastolic diameter; LVSD: left ventricular systolic diameter; LVWT: posterior wall shortening velocity; FAC: fractional area change; IRT/RR^0.5^: isovolumetric relaxation time normalized to the heart rate. Data are expressed as mean ± SD or medians (including the lower quartile and upper quartile). ^∗^*P* < 0.05 versus S; ^#^*P* < 0.05 versus C.

**Table 2:** Isolated heart data.

Variables	S (*n* = 8)	C (*n* = 8)	HIT (*n* = 8)
+dP/dt (mmHg/s)	1825 ± 496	1541 ± 557	1750 ± 337
–dP/dt (mmHg/s)	1375 ± 423	1208 ± 437	1200 ± 357
SP (mmHg)	100 ± 23.5	80 ± 12.1	93 ± 14.1

S: sedentary control animals; C: continuous moderate intensity training; HIT: high-intensity interval training; +dP/dt: maximum LV pressure development rate; –dP/dt: maximum LV pressure decrease rate; SP: systolic pressure. Data are expressed as mean ± SD. *P* > 0.05.

**Table 3:** Cardiac oxidative stress enzyme activity.

Variables	S (*n* = 8)	C (*n* = 8)	HIT (*n* = 8)
LH (nmol/mg of tissue)	251 ± 30.9	249 ± 55.7	186 ± 39.9^∗^^#^
CAT (*μ*mol/g)	80.4 ± 11.5	86.5 ± 9.7	119 ± 15.7^∗^^#^
GSH-Px (nmol/mg)	22.5 ± 4.9	33.1 ± 2.9^∗^	41.1 ± 7.7^∗^^#^

S: sedentary control animals; C: continuous moderate intensity training; HIT: high-intensity interval training; LH: lipid hydroperoxide; CAT: catalase; GSH-Px: glutathione peroxidase. Data are expressed as mean ± SD. ^∗^*P* < 0.05 versus S; ^#^*P* < 0.05 versus C.

**Table 4:** Cardiac energy metabolism.

Variables	S (*n* = 8)	C (*n* = 8)	HIT (*n* = 8)
Pyruvate dehydrogenase complex (nmol/g)	198 ± 39.5	165 ± 20.1	217 ± 35.4^#^
Lactate dehydrogenase (nmol/g)	101 ± 9.4	89.6 ± 9.5^∗^	80.4 ± 8.0^∗^
*β*-Hydroxyacyl coenzyme-A dehydrogenase (nmol/mg)	18.1 ± 4.1	19.5 ± 4.6	24.2 ± 4.8^∗^^#^
Citrate synthase (nmol/g)	27.0 ± 7.0	35.4 ± 9.0	74.7 ± 9.9^∗^^#^
Complex I (NADH dehydrogenase) (nmol/mg)	3.2 (1.8-4.1)	5.1 (4.3-6.4)^∗^	5.1 (3.9-6.0)^∗^
ATP synthase (nmol/mg)	33.5 ± 7.2	60.1 ± 8.1^∗^	61.8 ± 6.8^∗^

S: sedentary control animals; C: continuous moderate intensity training; HIT: high-intensity interval training. Data are expressed as mean ± SD or medians (including the lower quartile and upper quartile). ^∗^*P* < 0.05 versus S; ^#^*P* < 0.05 versus C.

## Data Availability

The authors confirm that the data supporting the findings of this study are available within the article.

## References

[1] Pfeffer M. A., Braunwald E. (1990). Ventricular remodeling after myocardial infarction: experimental observations and clinical implications. *Circulation*.

[2] Zornoff L. A. M., Paiva S. A. R., Duarte D. R., Sparado J. (2009). Ventricular remodeling after myocardial infarction: concepts and clinical implications. *Arquivos Brasileiros de Cardiologia*.

[3] Cohn J. N., Ferrari R., Sharpe N. (2000). Cardiac remodeling- concepts and clinical implications: a consensus paper from an international forum on cardiac remodeling. *Journal of the American College of Cardiology*.

[4] Azevedo P. S., Polegato B. F., Minicucci M. F., Paiva S. A., Zornoff L. A. (2016). Cardiac remodeling: concepts, clinical impact, pathophysiological mechanisms and pharmacologic treatment. *Arquivos Brasileiros de Cardiologia*.

[5] Guizoni D. M., Oliveira-Junior S. A., Noor S. L. R. (2016). Effects of late exercise on cardiac remodeling and myocardial calcium handling proteins in rats with moderate and large size myocardial infarction. *International Journal of Cardiology*.

[6] Daliang Z., Lifang Y., Hong F. (2019). Netrin-1 plays a role in the effect of moderate exercise on myocardial fibrosis in rats. *PLoS One*.

[7] Garza M. A., Wason E. A., Cruger J. R., Chung E., Zhang J. Q. (2019). Strength training attenuates post-infarct cardiac dysfunction and remodeling. *The Journal of Physiological Sciences*.

[8] Donniacuo M., Urbanek K., Nebbioso A. (2019). Cardioprotective effect of a moderate and prolonged exercise training involves sirtuin pathway. *Life Sciences*.

[9] Naderi N., Hemmatinafar M., Gaeini A. A. (2019). High-intensity interval training increase GATA4, CITED4 and c-Kit and decreases C/EBP*β* in rats after myocardial infarction. *Life Sciences*.

[10] Batista D. F., Gonçalves A. F., Rafacho B. P. (2013). Delayed rather than early exercise training attenuates ventricular remodeling after myocardial infarction. *International Journal of Cardiology*.

[11] Pfeffer J. M., Finn P. V., Zornoff L. A., Pfeffer M. A. (2000). Endothelin-A receptor antagonism during acute myocardial infarction in rats. *Cardiovascular Drugs and Therapy*.

[12] Paiva S. A. R., Novo R., Matsubara B. B. (2005). *β*-Carotene attenuates the paradoxical effect of tobacco smoke on the mortality of rats after experimental myocardial infarction. *Journal of Nutrition*.

[13] Minicucci M. F., Azevedo P. S., Martinez P. F. (2011). Critical infarct size to induce ventricular remodeling, cardiac dysfunction and heart failure in rats. *International Journal of Cardiology*.

[14] Moreira J. B. N., Bechara L. R. G., Bozi L. H. M. (2013). High- versus moderate-intensity aerobic exercise training effects on skeletal muscle of infarcted rats. *Journal of Applied Physiology*.

[15] Azevedo P. S., Minicucci M. F., Chiuso-Minicucci F. (2010). Ventricular remodeling induced by tissue vitamin A deficiency in rats. *Cellular Physiology and Biochemistry*.

[16] Minicucci M. F., Azevedo P. S., Oliveira Jr S. A. (2010). Tissue vitamin A insufficiency results in adverse ventricular remodeling after experimental myocardial infarction. *Cellular Physiology and Biochemistry*.

[17] Silva R. A. C., Gonçalves A. F., dos Santos P. P. (2017). Cardiac remodeling induced by all-trans retinoic acid is detrimental in normal rats. *Cellular Physiology and Biochemistry*.

[18] Assalin H. B., Rafacho B. P., Santos P. P. . (2013). Impact of the length of vitamin d deficiency on cardiac remodeling. *Circulation Heart Failure*.

[19] Gonçalves A. F., Polegato B. F., Fernandes A. A. (2018). Zinc supplementation attenuates cardiac remodeling after experimental myocardial infarction. *Cellular Physiology and Biochemistry*.

[20] Gaudron P., Hu K., Schamberger R., Budin M., Walter B., Ertl G. (1994). Effect of endurance training early or late after coronary artery occlusion on left ventricular remodeling, hemodynamics, and survival in rats with chronic transmural myocardial infarction. *Circulation*.

[21] Jain M., Liao R., Ngoy S., Whittaker P., Apstein C. S., Eberli F. R. (2000). Angiotensin II receptor blockade attenuates the deleterious effects of exercise training on post-MI ventricular remodelling in rats. *Cardiovascular Research*.

[22] Kloner R. A., Kloner J. A. (1983). The effect of early exercise on myocardial infarct scar formation. *American Heart Journal*.

[23] Hammerman H., Schoen F. J., Kloner R. A. (1983). Short-term exercise has a prolonged effect on scar formation after experimental acute myocardial infarction. *Journal of the American College of Cardiology*.

[24] de Waard M. C., van der Velden J., Bito V. (2007). Early exercise training normalizes myofilament function and attenuates left ventricular pump dysfunction in mice with a large myocardial infarction. *Circulation Research*.

[25] Bito V., de Waard M. C., Biesmans L. (2010). Early exercise training after myocardial infarction prevents contractile but not electrical remodelling or hypertrophy. *Cardiovascular Research*.

[26] Xu X., Wan W., Ji L. (2008). Exercise training combined with angiotensin II receptor blockade limits post-infarct ventricular remodelling in rats. *Cardiovascular Research*.

[27] Xu X., Wan W., Powers A. S. (2008). Effects of exercise training on cardiac function and myocardial remodeling in post myocardial infarction rats. *Journal of Molecular and Cellular Cardiology*.

[28] Martinez P. F., Bonomo C., Guizoni D. M. (2016). Modulation of MAPK and NF-KB signaling pathways by antioxidant therapy in skeletal muscle of heart failure rats. *Cellular Physiology and Biochemistry*.

[29] Martinez P. F., Bonomo C., Guizoni D. M. (2015). Influence of N-acetylcysteine on oxidative stress in slow-twitch soleus muscle of heart failure rats. *Cellular Physiology and Biochemistry*.

[30] Azevedo P. S., Minicucci M. F., Santos P. P., Paiva S. A. R., Zornoff L. A. M. (2013). Energy metabolism in cardiac remodeling and heart failure. *Cardiology in Review*.

